# Sex Differences in Heart Failure: What Do We Know?

**DOI:** 10.3390/jcdd10070277

**Published:** 2023-06-29

**Authors:** Allegra Arata, Fabrizio Ricci, Mohammed Y. Khanji, Cesare Mantini, Francesco Angeli, Roberta Aquilani, Angela Di Baldassarre, Giulia Renda, Anna Vittoria Mattioli, Savina Nodari, Sabina Gallina

**Affiliations:** 1 Department of Neuroscience, Imaging and Clinical Sciences, “G. d’Annunzio” University of Chieti-Pescara, 66100 Chieti, Italy; allegra.arata@studenti.unich.it (A.A.); cesare.mantini@gmail.com (C.M.); giulia.renda@unich.it (G.R.); sabina.gallina@unich.it (S.G.); 2 William Harvey Research Institute, NIHR Barts Biomedical Research Centre, Queen Mary University London, Charterhouse Square, London EC1M 6BQ, UK; m.khanji@qmul.ac.uk; 3 Department of Clinical Sciences, Lund University, 214 28 Malmö, Sweden; 4 Department of Medical and Surgical Sciences, University of Bologna, Via Zamboni, 33-40126 Bologna, Italy; francesco.angeli6@studio.unibo.it; 5 Cardiac Surgery Intensive Care Unit, Heart Department, SS Annunziata University Hospital, Via dei Vestini 5, 66100 Chieti, Italy; roberta_aquilani@yahoo.it; 6 Department of Medicine and Aging Sciences, and Reprogramming and Cell Differentiation Lab, Center for Advanced Studies and Technology (CAST), “G. d’Annunzio” University of Chieti-Pescara, 66100 Chieti, Italy; angela.dibaldassarre@unich.it; 7 Surgical, Medical and Dental Department of Morphological Sciences Related to Transplant, Oncology and Regenerative Medicine, University of Modena and Reggio Emilia, 41100 Modena, Italy; annavittoria.mattioli@unimore.it; 8 Department of Cardiology, University of Brescia and ASST “Spedali Civili” Hospital, 25123 Brescia, Italy; savina.nodari@unibs.it

**Keywords:** sex, gender medicine, heart failure, menopause, pregnancy, guidelines, treatment

## Abstract

**Highlights:**

- Women predominantly exhibit HFpEF compared to men.
- Factors exclusive to women, such as adverse pregnancy outcomes and premature menopause, elevate the risk of HF.
- The establishment of sex-specific optimal drug dosages and concrete guidelines for device therapy is essential.
- Concerted multidisciplinary initiatives are crucial to bridge the existing sex disparities in HF management.

**Abstract:**

Heart failure (HF) remains an important global health issue, substantially contributing to morbidity and mortality. According to epidemiological studies, men and women face nearly equivalent lifetime risks for HF. However, their experiences diverge significantly when it comes to HF subtypes: men tend to develop HF with reduced ejection fraction more frequently, whereas women are predominantly affected by HF with preserved ejection fraction. This divergence underlines the presence of numerous sex-based disparities across various facets of HF, encompassing aspects such as risk factors, clinical presentation, underlying pathophysiology, and response to therapy. Despite these apparent discrepancies, our understanding of them is far from complete, with key knowledge gaps still existing. Current guidelines from various professional societies acknowledge the existence of sex-based differences in HF management, yet they are lacking in providing explicit, actionable recommendations tailored to these differences. In this comprehensive review, we delve deeper into these sex-specific differences within the context of HF, critically examining associated definitions, risk factors, and therapeutic strategies. We provide a specific emphasis on aspects exclusive to women, such as the impact of pregnancy-induced hypertension and premature menopause, as these unique factors warrant greater attention in the broader HF discussion. Additionally, we aim to clarify ongoing controversies and knowledge gaps pertaining to the pharmacological treatment of HF and the sex-specific indications for cardiac implantable electronic devices. By shining a light on these issues, we hope to stimulate a more nuanced understanding and promote the development of more sex-responsive approaches in HF management.

## 1. Introduction

HF emerges as an important contributor to global morbidity and mortality in both men and women, despite advancements in medical and device-based treatments. It is responsible for 35% of cardiovascular-related deaths in women [1]. Guidelines on HF across Europe, America, and Japan acknowledge the existence of sex disparities among patients with HF. Over the years, numerous studies have underscored significant sex-based distinctions in various facets of HF: its epidemiology, risk factors, pathophysiology, clinical manifestations, associated comorbidities, triggers for decompensation, diagnostic processes, causes of death, treatment approaches, and outcomes (Table 1).

Despite these findings, the persistent underrepresentation of women in these studies presents a significant challenge, leaving many questions about sex-based differences in HF unanswered [17,18,19,20]. Numerous critical issues that directly affect the health outcomes of women with HF remain unresolved [21,22,23]. As a result, guidelines offer only scant advice on sex-specific recommendations (Table 2).

Epidemiologically, the lifetime risk of developing HF is approximately the same for both sexes. Women represent approximately 25% of patients diagnosed with HF with reduced ejection fraction (HFrEF). In contrast, they make up about 50% of those affected by HF with preserved ejection fraction (HFpEF) [24].

## 2. Heart Failure: Definition and Phenotypes of Disease

The universal definition defines HF as a clinical syndrome with symptoms or signs caused by cardiac structural and/or functional abnormality and corroborated by elevated natriuretic peptide levels or observable evidence of pulmonary or systemic congestion [25]. The clinical classification of HF has historically relied on left ventricular ejection fraction (LVEF), with traditionally uniform LVEF thresholds for both sexes, even though sex-specific criteria exist for other cardiac characteristics such as left ventricular hypertrophy.

The classification of HF into specific phenotypes (reduced, mildly reduced, and preserved ejection fraction) is key for clinical decision-making, particularly regarding treatment strategies. Imaging studies have shown that women typically exhibit higher LVEF than men [3,4,26,27,28,29]. This can be primarily attributed to a higher stroke volume per given end-diastolic volume, potentially indicative of intrinsic sex differences in cardiac function or responses to hemodynamic stress. This variation is independent of potential confounding variables like age, blood pressure, heart rate, body size, and cardiovascular risk factors as shown in the Dallas Heart Study [5]. These observations underscore the need for sex-specific criteria in clinical decisions, advocating for an LVEF threshold potentially up to 3 percentage points higher in women than in men [29]. Furthermore, the findings imply that women with LVEF on the lower limits of the normal range might be experiencing significant cardiac dysfunction. This could elucidate their tendency to develop symptomatic HF despite having what is conventionally considered as preserved LVEF.

Current guidelines recommend the initial measurement of NPs levels either to establish a HF diagnosis or to rule it out in symptomatic patients. Additionally, NP measurement is recommended for risk stratification and prognosis in chronic or hospitalized HF patients. ESC and AHA/ACC/HFSA guidelines define plasma concentration of NPs cutoff as BNP >35 pg/mL or NT-proBNP >125 pg/mL as suggestive of HF. Emerging evidence suggests that sex-based differences in NP levels exist. Estrogens, which are known to stimulate the NP system, might explain the observed NP excess in women [30]. Conversely, androgens may inhibit the NP system, contributing to lower NP levels in men and possibly explaining their lack of cardiovascular protection relative to women. Suthahar et al. found that while the majority of biomarkers were robustly associated with incident HF in both sexes, subtle sexes-related differences were observed in the prognostic value of individual biomarkers [6]. Interestingly, post-menopausal women with HFpEF appear to have lower NP levels compared to men. Emerging data suggests the need to define sex and hormonal status-related thresholds for circulating NPs. However, due to limited data on women with HF, current guidelines apply the same NP threshold for both sexes.

## 3. Sex-Specific Risk Factors for Heart Failure

Traditional cardiovascular risk factors, including obesity, diabetes, smoking, arterial hypertension, and dyslipidemia, contribute to an increased longitudinal risk of HF in both men and women. As previously stated, women are predisposed to HFpEF, whereas men predominate in HFrEF. Important sex-based differences in the impact of traditional risk factors exist and seem to play a role in the pathophysiological explanation of phenotypic differences between women and men. Diabetes and obesity are independently associated with the risk of HF, but to a greater extent in women compared with men. Diabetes has been shown to be a more potent risk factor for the development of HF in women than in men (5-fold higher risk in women vs. 2-fold higher risk in men) [31]. Furthermore, recent data have demonstrated important sex differences in response to glucose intolerance and insulin resistance, with greater increases in LV mass observed among women [32,33]. Globally, obesity is more prevalent in women than men (11.5% vs. 6.9%) [34], and its association with HF risk is greater for women. Underlying mechanisms seem to be related to increases in blood volume coupled with limited ventricular distensibility, mediated at least in part by excess adipose tissue-derived signaling molecules, including neprilysin and aldosterone [35]. Moreover, central obesity is more prevalent among women after menopause [36,37]. This form of obesity is associated with the deterioration of cardiac function even among individuals with normal BMI. These conditions are commonly associated with HFpEF. Obesity affects up to half and diabetes mellitus affects approximately 45% of patients with HFpEF supporting the inflammatory paradigm of HFpEF. Coronary microvascular dysfunction and endothelial inflammation play a key role in HFpEF in contrast to HFrEF to their predisposition to macrovascular coronary artery disease and myocardial infarction.

Conversely, women reportedly have lower prevalence rates of hypertension and smoking compared to men, both of which are associated with a higher risk of coronary artery disease and myocardial infarction–direct precursors to the HFrEF phenotype. Nevertheless, it is worth noting that smoking rates among women, particularly young women, are on the rise. This deserves heightened attention from the scientific community given that smoking is also a risk factor for coronary artery vasospasm and peripartum cardiomyopathy (PPCM). 

Furthermore, there are non-traditional cardiovascular risk factors exclusive to women that become particularly pertinent at different stages of life which could partially account for the greater prevalence of HFpEF in women [8]. Although many studies have delved into the relationship between adverse pregnancy outcomes and premature menopause in the development of HF in women, the exact underlying pathophysiological mechanisms are not yet fully understood and warrant further investigation.

Hypertensive disorders of pregnancy (HDP), for instance, are associated with short-term aberrations in cardiac structure and function. Notably, women who have had HDPs display pronounced differences in left ventricular structure and function even ten years post-pregnancy [7]. Conditions like preeclampsia and eclampsia are recognized as independent risk factors for future HF hospitalizations, specifically HFpEF. Considering the established connection between HDP and an elevated risk of HF later in life, these conditions may be classified as risk-enhancing factors, serving as essential markers for targeting women in HF prevention strategies. 

Anemia and iron deficiency are common in HF and are associated with worse symptoms and outcomes in HF patients. Commonly, anemia has been defined as hemoglobin levels of <12 g/dL in women and <13 g/dL in men. Several reports suggest a marked female predominance in the prevalence of anemia and iron deficiency in the setting of HF [38,39,40]. Particularly, it has been noted that the percentage of women rises as anemia severity increases in these patients. Anemia in HF is considered to develop due to a complex interaction of impaired iron metabolism, blunted erythropoietin production and response, kidney disease, altered renin-angiotensin system activity, and systemic inflammation, although micronutrient insufficiency and blood loss may contribute [41]. Overall, whereas the association of anemia and HF has been unequivocally associated with a worse prognosis compared with HF alone, the triggers and mechanisms that underlie this association remain often speculative and thus not yet amenable to specific interventions. Anemia can exacerbate cardiac dysfunction due to the resultant cardiac stress from tachycardia and increased resting stroke volume, as well as reduced renal blood flow causing fluid retention, and further straining the heart. This condition can trigger a self-perpetuating cycle where chronic HF induces anemia, which in turn worsens HF and damages the kidneys, escalating both conditions. This harmful cycle is referred to as the cardio-renal-anemia syndrome [42].

The cardio-protective role of estrogen is well-acknowledged. Guidelines from the American College of Cardiology/American Heart Association (ACC/AHA) and the European Society of Cardiology have categorized premature menopause (occurring before the age of 40) and HDP as risk-enhancing factors for atherosclerosis. A large cohort study involving 28,516 women concluded that both nulliparity and shorter total reproductive duration were associated with a higher risk of incident HF [9]. The causal relationship between HF development and estrogen deficiency, commonly associated with menopause, is not yet definitively established. However, current hypotheses suggest it may be linked to the modulation of endothelial nitric oxide synthase (eNOS). Estrogen is known to upregulate eNOS and activate phosphoinositide 3-kinase signaling, which in turn leads to further eNOS activation [43]. The resulting surge in nitric oxide production may have significant physiological implications. As estrogen levels decline with age, this might contribute to an increased risk of HF over time [44]. Future research is anticipated to shed more light on these potential mechanisms and their role in explaining the observed gender-based differences in cardiovascular risk. At present, there is not sufficient evidence to endorse hormone replacement therapy and selective estrogen-receptor modulators for primary or secondary cardiovascular prevention (Class III, Level of Evidence A). Nevertheless, a deeper understanding of the mechanisms associated with premature menopause could be instrumental in shaping effective HF prevention strategies.

Moreover, it is important to note the influence of mental health and psychosocial conditions on cardiovascular outcomes. Stress, anxiety, depression, and adverse psychosocial circumstances have been linked to worse cardiovascular outcomes [45]. The ACC/AHA Guidelines on HF indicate that these non-traditional cardiovascular risk factors might contribute more significantly to the incidence of HF in women than in men, highlighting the importance of a holistic approach to prevention and treatment.

## 4. Sex-Specific Differences in Ischemic and Non-Ischemic Cardiomyopathies

### 4.1. Ischemic Heart Disease

Ischemic heart disease (IHD) is the primary cause of death and one of the most common causes of HF in women worldwide. It has become evident that both biological and socio-environmental factors play crucial roles in women’s health. This understanding has led to important insights into the expanded spectrum of IHD in women, including obstructive coronary artery disease, coronary microvascular dysfunction, and endothelial dysfunction. Despite progress in understanding and managing IHD, sex-specific disparities remain in terms of disease outcomes, risk factors, clinical presentation, diagnostic workup, and disease management.

Men and women share many traditional risk factors for IHD, but additional sex-specific risk factors have been identified [46]. These include menopause, which marks a significant cardiovascular transition due to a loss of estrogen, adversely affecting arterial function and the cholesterol profile. This transition increases the prevalence of metabolic syndrome and truncal obesity [46,47]. Other risk factors such as hypertension, diabetes mellitus, and smoking pose a greater threat to women than men. Additionally, conditions unique to women like early menopause, gestational diabetes mellitus, hypertension, preeclampsia and eclampsia during pregnancy, and systemic inflammatory disorders significantly increase a woman’s risk for IHD [48,49,50]. 

Women’s presentation of IHD can differ significantly from men’s. Fewer women experience the classic symptoms of chest pain, and many instead exhibit symptoms such as dyspnea, weakness, arm, back or jaw pain, palpitations, light-headedness, or loss of appetite [51,52]. The pathophysiology of IHD also varies between the sexes, with women exhibiting a higher prevalence of non-obstructive coronary disease and plaque erosion, compared to plaque rupture in men [53,54,55]. 

Diagnostic imaging plays a crucial role in detecting IHD, particularly in women, who often show vascular dysfunction in the absence of obstructive coronary disease. Despite the proven efficacy of revascularization and guideline-recommended pharmacotherapy, women are less likely than men to receive these interventions. Moreover, women are less likely to be referred and participate in cardiac rehabilitation following a myocardial infarction [56,57,58].

Despite a decline in IHD mortality in women over the past four decades, sex-based differences persist in terms of outcomes. Women presenting with acute coronary syndrome have less obstructive disease but exhibit higher in-hospital and 1-year mortality compared to men. They also experience higher incidences of cardiogenic shock and HF. Significant disparities persist in timely revascularization, particularly in young women [59,60,61]. Additionally, several subgroups, especially ethnic minority groups and young women, show a greater disparity in outcomes.

### 4.2. Dilated Cardiomyopathy

Epidemiological studies indicate that the burden of dilated cardiomyopathy (DCM) in the population is lower in women. In Olmsted County, for instance, the prevalence of DCM was observed to be 19.4 per 100,000 in women vs. 58.0 per 100,000 in men [62]. This trend is consistent across patient cohorts, with women making up only 31% and 33% of patients undergoing cardiac transplantation for DCM and those registered in a modern DCM registry, respectively [63,64]. However, this sex discrepancy does not appear in pediatric DCM cases [65]. These findings might reflect differences in healthcare access among adult women or actual biological variations in DCM manifestations between children and adults. 

Numerous factors can potentially lead to DCM, including coronary artery disease, infections, tachyarrhythmias, and various environmental influences. The complex relationship between biology and environment is evident in acquired DCM, with alcoholic cardiomyopathy being more common in men due to their higher alcohol consumption [66]. Despite this, women are more susceptible to the harmful effects of alcohol and develop alcoholic cardiomyopathy with less alcohol intake. Additionally, men are hospitalized for myocarditis twice as often as women, but women’s mortality rate doubles upon hospitalization [67]. 

When the cause of DCM remains elusive, it is referred to as “idiopathic” DCM, defined as the presence of both LV dilatation and systolic dysfunction but without evidence of ischemic or known causes, except genetic. Interestingly, about a third to a half of DCM cases are either familial or genetic [68]. Over 50 putative DCM-related genes have been identified, but only 19 genes account for the majority of DCM cases [69,70]. The success rate of identifying DCM through genetic testing in both adult and child probands is around 30%, with no variation based on sex. 

Although women and men are equally likely to be diagnosed with DCM, the relationship between gender and the expression of pathogenic gene variants in DCM remains incompletely understood. 

Titin truncating variants, the most common identifiable cause of DCM, show higher penetrance and younger presentation in men, possibly due to higher alcohol abuse. However, women with these variants have a higher risk of peripartum DCM.

Dilated cardiomyopathy (DCM) has a genetic origin or is familial in about 40% of cases. Both genders are equally susceptible to DCM, with the disease following various modes of inheritance such as X-linked, recessive, and matrilineal (involving variations in mitochondrial genes). However, autosomal dominant inheritance is the most common.

Other sarcomeres’ gene mutations, such as β-myosin heavy chain (MYH7), cardiac troponin-T (TNNT2), α-tropomyosin (TPM1), and cardiac troponin-C (TNNC1) genes, cause DCM across all ages, with no clear sex-based differences [64,65]. Lamin A/C gene (LMNA) mutations present with various symptoms, but women with LMNA heart disease are less likely to have life-threatening ventricular arrhythmias, suggesting male sex is a risk factor [71].

Despite these observations, there are few consistent sex-related trends in DCM development, highlighting the complicated interplay between biological and societal/environmental influences. Genetic testing and genotyped registries are valuable tools for understanding these relationships and improving management and risk prediction.

### 4.3. Peripartum Cardiomyopathy 

PPCM is a potentially life-threatening condition, characterized as idiopathic cardiomyopathy presenting either towards the end of pregnancy or in the months following delivery, miscarriage, or abortion, in the absence of any other identifiable causes for HF. The condition is typically marked by an LVEF of less than 45%. However, diagnosis may still apply to those with EF values between 45% and 50% if they exhibit other distinctive features of PPCM.

The incidence of PPCM varies significantly based on women’s ethnic and regional backgrounds, with a higher prevalence noted in Africans and African Americans. In recent years, an increase in incidence has been observed among the population in the USA and Asia.

Several risk factors have been associated with PPCM, including multiparity, multiple pregnancies, family history, ethnicity, smoking, diabetes, hypertension, pre-eclampsia, nutritional deficiencies, viral myocarditis, age (with older mothers being at greater risk), and autoimmune processes. However, the etiology of PPCM remains multifactorial, not fully understood, and thought to be quite heterogeneous. It potentially involves various pregnancy-related factors, such as hemodynamic, angiogenic, metabolic, hormonal, and oxidative stress factors.

Early diagnosis of PPCM, achieved through a comprehensive assessment of a patient’s medical history, physical examination, electrocardiogram (ECG), BNP assessment, and various cardiac imaging techniques, coupled with the prompt initiation of HF medications, has been associated with improved patient outcomes [72]. Nevertheless, further research is needed to establish concrete recommendations regarding subsequent pregnancies and breastfeeding for PPCM patients.

### 4.4. Hypertrophic Cardiomyopathy

Hypertrophic cardiomyopathy (HCM) presents differently between sexes, according to various large-scale studies. Women represent 35–45% of HCM patients and are typically diagnosed 6–9 years later than men, often with more severe symptoms [62,73,74,75]. Women also present with lower exercise capacity and greater diastolic dysfunction and pulmonary hypertension [73,76]. Moreover, women with HCM have smaller LV cavities, more LV outflow tract obstruction, and increased use of specific treatments compared to men. In terms of progression, women have a higher risk of HF progression, stroke, and atrial fibrillation [77], although the incidence of sudden cardiac death is similar for both sexes [70,73,74,75]. Women also have a higher all-cause mortality rate, even after adjusting for various factors. Genetic testing shows that a higher percentage of women carry sarcomere gene variants, with variable effects on disease presentation and progression based on the specific gene variant. In a nationwide family risk study, researchers observed that the relatives of female patients with HCM are more likely to be affected by the disease compared to the relatives of affected males [78]. This finding suggests the possibility of a multifactorial threshold model of inheritance, in which female individuals carry a higher genetic load for HCM. According to this model, females would require a greater number of, or more potent, susceptibility genes than males to inherit and express the disease phenotype. Consequently, females would be more likely to transmit the disease to their offspring and have siblings with the disease. This phenomenon is referred to as the Carter effect [78]. This suggests that the genetic factors contributing to HCM susceptibility may differ between males and females, with females requiring a higher genetic burden to manifest the disease.

### 4.5. Fabry Disease

Fabry Disease (FD) is an X-linked lysosomal storage disorder, caused by mutations in the GLA gene, leading to a deficiency in α-galactosidase A enzyme activity. This deficiency causes an accumulation of glycosphingolipids, primarily globotriaosylceramide, in lysosomes across various cell types, including cardiomyocytes, conduction system cells, fibroblasts, as well as endothelial and smooth muscle vascular cells. Consequently, this accumulation promotes ventricular hypertrophy and fibrosis, contributing to the onset of HF, valve disease, angina, arrhythmias, cardiac conduction abnormalities, and potentially sudden death. FD is diagnosed in approximately 0.9% of patients with hypertrophic cardiomyopathy. The main cardiac manifestation of FD is left ventricular hypertrophy, which tends to develop earlier and progress more rapidly in men than women, due to lyonization. Accordingly, female carriers can exhibit a wide range of symptoms due to the random nature of X-inactivation, as some cells will express the normal gene while others will express the mutated version, leading to variability in the clinical manifestation of the disease. This variability in expression due to lyonization is sometimes referred to as “manifesting heterozygote” [79,80,81].

Left ventricular hypertrophy, along with fibrosis, results in systolic and diastolic dysfunction, which in combination with valve disease, myocardial ischemia, and conduction disorders, increases the risk of HF in FD. Although HF is more common in men with FD, women tend to have a longer mean survival rate free from HF. Several therapeutic interventions, targeting the enzymatic defect or reducing substrate accumulation, have shown promise in reducing or stabilizing left ventricular mass and wall thickness, and in reducing the incidence and delaying the onset of clinical events, such as HF. However, the sex-specific effectiveness of these treatments on major cardiovascular endpoints, such as cardiovascular mortality, HF, arrhythmias, or the need for a cardiac device, is not yet well established. 

### 4.6. Cardiac Amyloidosis

Cardiac amyloidosis, characterized by extracellular amyloid fibrils in the heart, is predominantly seen in men, who make up 90% of patients with wild-type transthyretin cardiac amyloidosis (ATTRwt) [82]. Sex hormones may influence this condition, as animal models show that 5α-dihydrotestosterone raises transthyretin expression more effectively than estradiol [83]. Women with ATTRwt are typically diagnosed later and have a more advanced disease, which may be due to diagnostic delays and a lack of sex-specific diagnostic standards. Male predominance is also evident in hereditary transthyretin cardiac amyloidosis, particularly in late-onset cases [84]. Men seem to have greater myocardial involvement, potentially due to differences in fibril composition.

Light chain amyloidosis (AL) incidence seems less influenced by sex, with only a slightly higher incidence in men [85]. Women with hereditary transthyretin cardiac amyloidosis and AL generally do not show significant clinical differences from men at baseline. No differences in all-cause mortality between sexes have been reported in AL, ATTRwt, and hereditary transthyretin cardiac amyloidosis [86,87].

### 4.7. Arrhythmogenic Cardiomyopathy

Arrhythmogenic cardiomyopathy, characterized by fibro-fatty replacement of the myocardium leading to electrical instability and ventricular dysfunction, is typically inherited in an autosomal dominant manner and primarily affects genes encoding desmosomal proteins. This condition has variable penetrance, expressivity, and a high risk of life-threatening ventricular arrhythmias [88]. Men are more frequently affected, presenting with abnormal ECGs, late potentials, worse biventricular cardiac function, and a higher risk for ventricular arrhythmias and HF compared to women [89,90,91].

Sex hormones could influence this difference, with high testosterone levels exacerbating and normal estradiol levels decreasing cardiomyocyte apoptosis and lipogenesis [92]. Furthermore, vigorous-intensity exercise training might also contribute to arrhythmias and cardiomyopathy progression in arrhythmogenic cardiomyopathy, potentially due to the historically higher participation of men in competitive sports [93].

### 4.8. Inflammatory Cardiomyopathies

Inflammatory cardiomyopathies can be idiopathic, autoimmune, or infectious in origin. They are linked with increased mortality due to cardiovascular diseases in both sexes. However, the prevalence of systemic autoimmune disorders like rheumatoid arthritis (RA) and systemic lupus erythematosus (SLE) is sex-dependent, being more common in women [94]. These conditions frequently induce organ and tissue dysfunction, with myocardial microvasculature being particularly affected, leading to the development of inflammatory cardiomyopathies and expedited atherosclerotic diseases in women. Multiple studies have identified higher risk and mortality from HFpEF in patients with RA compared to the general population [95,96,97]. Chronic inflammation is a known driver of HF in RA, but further research is needed to elucidate the disease course, imaging findings, biomarker evaluations, and potential treatments for HF in the context of autoimmune diseases.

## 5. Heart Failure Management

HF management has emerged as a key focus in recent years. It has been observed that women diagnosed with HF tend to have better survival rates than men. The OPTIMIZE-HF registry documented a lower 1-year mortality rate for women suffering from acute HF, even though they are more likely to miss out on optimal guideline-directed medical therapy (GDMT). Evidence-based treatment for HFrEF has been well-established, while therapy for HFpEF primarily aims at alleviating symptoms and aggressively managing risk factors and other comorbidities in accordance with clinical guidelines. Recent large RCTs have demonstrated the benefits of sodium-glucose co-transporter-2 inhibitors (SGLT2i) across a broad spectrum of LVEF in HF patients [98,99]. The European Society of Cardiology guidelines now recommend these drugs for both the treatment and prevention of HFrEF, with the highest level of evidence and recommendation. Future updates regarding their use in HFpEF are anticipated.

Historically, GDMT for HF has been the same for both sexes, despite the lack of specific evidence to support this uniform approach. Studies evaluating sex differences in drug efficiency for HF patients have been limited, due to the underrepresentation of women in all stages of drug development and a lack of sex-specific evaluation of drug efficacy and safety in RCTs, which were predominantly conducted on middle-aged men. Despite these limitations, the idea that HF therapy might benefit from a sex-specific approach is gaining traction. There are known sex differences in pharmacokinetics due to physiological variations in body composition, organ function, hormonal changes (such as menopause or hormone replacement therapy), and drug absorption, distribution, metabolism, and excretion [100,101,102,103,104,105].

Recent studies suggest that women with HFrEF may require lower doses of renin-angiotensin-aldosterone system inhibitors and β-blockers than men, prompting a discussion on the definition of optimal medical therapy for each sex. Notably, hospitalization rates show no sex difference for patients treated with SGLT2i, and benefits are observed equally in both men and women [10]. Nevertheless, a subgroup analysis of the EMPA-REG OUTCOME trial indicates potential sex differences for overall and cardiovascular mortality. Furthermore, a meta-regression analysis of 26 trials suggested a progressive decrement in benefit in women, as the percentage of women included in the SGLT2i arm exceeded 50% [14].

In a pre-specified sex subgroup analysis of the PARAGON-HF trial, a more favorable effect of the angiotensin receptor-neprilysin inhibitor compound was observed in women with HF and LVEF not lower than 60% compared to men at similar LVEF [106]. However, no sex differences were observed in the PARADIGM-HF trial for HFrEF patients, where less than a quarter of the patients were women [11]. Similarly, the TOPCAT trial did not show a significant interaction between treatment with mineralocorticoid receptor antagonists and sex [107].

Notably, when GDMTs are prescribed at equivalent doses, women tend to experience more adverse drug reactions (ADRs), which are often more severe than in men [102,108]. A large retrospective study from the Netherlands reported that 66% of serious ADRs leading to hospitalization occurred in women, with the most pronounced sex differences reported with diuretics and cardiac glycosides [15]. These findings can be attributed to higher exposure and reduced excretion of these drugs in women [109,110,111]. Higher rates of ADRs in women can negatively affect medication adherence, consequently reducing the long-term benefits of guideline-recommended HF drugs, leading to higher rates of drug discontinuation and hospitalizations compared to men. Despite the strong evidence of a higher incidence of ADRs in women, most HF trials do not report ADRs in a sex-disaggregated manner. A more comprehensive understanding of sex-related differences in ADRs and the underlying mechanisms could lead to more informed prescribing decisions and optimized therapy for HF.

Differences between men and women have also been observed regarding cardiac resynchronization therapy (CRT). Current guidelines restrict the strongest recommendation for CRT-D (Class IA) to patients with left bundle branch block (LBBB) and a QRS duration of 150 ms or longer, while those with a QRS of 120 to 149 ms receive a weaker recommendation (Class IIa). CRT benefits appear greater for women and occur at a lower QRS duration of 130 ms compared to 140 ms in men. An individual-patient-data meta-analysis revealed a 76% reduction in HF or death from CRT for women with no significant benefit in men at a QRS duration between 130 and 149 ms. Both sexes benefited from CRT with a QRS duration of 150 ms [16].

## 6. Does a Gender-Related Issue Exist in Heart Failure?

The decision to focus on sex-based differences, specifically referring to biological characteristics, in this study was motivated by the current limited understanding of gender-based disparities in HF research. The availability of clear and comprehensive reports regarding the epidemiology and outcomes of HF based on gender remains insufficient. The underrepresentation of women in clinical trials, and potential biases in healthcare provision need to be explored and addressed where disparities still exist. This comprehensive approach will contribute to a more nuanced understanding of the complex interplay between gender and HF. Additionally, the influence of hormone replacement or hormone-blocking agents (including post-menopausal hormone replacement therapy) on HF is not fully understood and requires further research [112]. 

## 7. Transformative Solutions for Improvement

**Figure 1:**
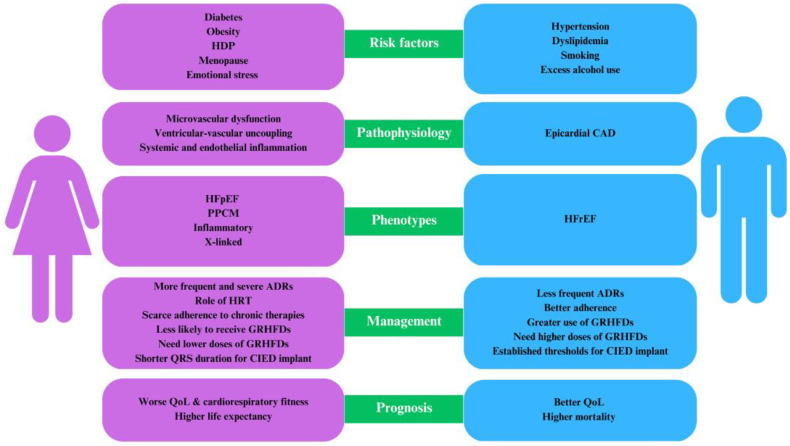
Clinical profiles of HFpEF in females vs. males. ADRs = adverse drug reactions; CAD = coronary artery disease; CIED = cardiac implantable electronic device; GRHFDs = guideline-recommended heart failure drugs; HFpEF = heart failure with preserved ejection fraction; HFrEF = heart failure with reduced ejection fraction; HPDs = hypertensive disorders; HTR = hormone replacement therapy; PPCM = peripartum cardiomyopathy; QoL = quality of life.

To address sex disparities and improve health outcomes, several transformative solutions have been proposed. These include fostering a greater understanding of the biological and sociocultural factors contributing to sex-specific differences in HF, advocating for the inclusion of women and underrepresented minorities in clinical research, implementing guidelines that account for sex-specific risk factors and presentation, and providing equitable access to diagnostic and therapeutic strategies. Improved patient education and empowerment could play a significant role. Encouraging women to understand their own cardiovascular risk and to engage actively in their healthcare decisions could help bridge the gap in outcomes and contribute to the reduction in sex-specific disparities in HF (Figure 1).

## 8. Conclusions

HF exhibits numerous sex-related differences across its various aspects, including prevalence, classification, risk factors, phenotype expression, management, and outcomes. Despite recent advancements in understanding and awareness in this field, the intricate molecular and pathophysiological mechanisms underlying sex-specific HF remain only partially elucidated. Overall, available data suggest that women are more prone to developing HFpEF, and sex-specific risk factors significantly contribute to the development of HF. Furthermore, disease management, particularly drug response and indications for cardiac resynchronization, also exhibit sex-specific differences. The impact of hormone replacement and antagonist agents on HF are not well studied. As a result, more concerted efforts are necessary to develop personalized treatment approaches for HF in both women and men. Currently, international guidelines generally lack sex-based recommendations for HF, primarily due to the underrepresentation of females in RCTs. To bridge the existing gaps in knowledge and ensure future evidence-based, sex-specific recommendations, it is imperative to include a larger number of women in clinical trials and screening programs and equitable healthcare provisions regardless of sex.

## Figures and Tables

**Table 1:** Sex-related differences in HF: summary of relevant evidence from the literature.

Study Cohort/ First Author Name	Country	Study Design	Year of Publication	Women, *n* (%)	Key Messages
The LIFE study [2]	Denmark, Finland, Iceland, Norway, Sweden, the United Kingdom, and the United States	Retrospective cohort study	2001	391 (41%)	Female sex is an independent predictor of higher systolic LV function in hypertensive patients with electrocardiographic LV hypertrophy.
Alfakih et al. [3]	United Kingdom	Retrospective cohort study	2003	30 (50%)	Sex differences in the LV and RV volumes measured by CMR were statistically significant apart from the LVEF.
Framingham Heart Study Offspring Cohort [4]	United States	RCT, post-hoc analysis	2002	79 (40%)	Cardiovascular magnetic resonance measures of LV volumes, mass, and linear dimensions differ significantly according to sex and body size. All unadjusted LV parameters were significantly greater in men than in women (*p* 0.001) but no significant differences in LVEF between men (69%) and women (70%).
The Dallas Heart Study [5]	United States	Prospective cohort study	2006	1435 (55%)	Women have higher LVEF than men, reflecting a higher stroke volume for a given EDV. LVEF was higher by 5% in women compared to men.
Suthahar et al. [6]	Netherlands, the United States	Retrospective cohort study	2020	12,087 (53%)	Subtle sex-related differences in the prognostic value of individual biomarkers.
Countouris et al. [7]	United States	Prospective cohort study	2021	132 (102, (77%) normotensive, 30 (22%) HPD history	Women with HDP are more likely to have evidence of increased LV wall thickness, remodeling, and abnormal diastolic function in the decade after pregnancy and thus require closer surveillance and early and targeted therapies for CVD prevention.
Williams et al. [8]	United States	Meta-analysis	2021	2,532,515 (100%) women were included in the study: 2,404,486 (94%) without and 128,029 (5%) with preeclampsia/eclampsia.	Preeclampsia/eclampsia is an independent risk factor for future hospitalizations for HfpEF.
Hall et al. [9]	United States	Retrospective cohort study	2017	28,516 (100%)	Premature menopause is associated with a higher risk of incident HF, and null parity is associated with a higher risk for incident HF with preserved ejection fraction.
BIOSTAT-CHF study [10]	Europe	Prospective study cohort	2019	1819 (24%)	β-blockers were more frequently used at baseline than were ACE inhibitors or ARBs, and more often in men than in women.
PARAGON-HF trial [11]	Europe, North America, Latin America, Asia-Pacific	RCT	2019	4796 (51.7%)	Even when HfpEF is the predominant phenotype in women, they were significantly less likely than men to be treated with a nitrate and an MRA.
Merrill et al. [12]	United States, Argentina, Brazil, Canada, Russia, Georgia	RCT, post-hoc analysis	2019	1767 (49.9%)	Women are significantly more likely to be taking calcium channel blockers, whereas men are more likely to be taking β-blockers.
Dewan et al. [13]	North America, Latin America, Europe, Russia, Asia-Pacific	RCT, post-hoc analysis	2019	15,415 (21%)	Women are more often treated with digitalis and ARBs, but less likely with an ACEI compared with men.
Mahmoud et al. [14]	United States	Meta-analysis	2017	58%	SGLT2i progressive decrement in benefit in women.
Rodenburg et al. [15]	Netherlands	Retrospective cohort study		14,207 (54%)	Serious ADRs occur more in women especially with diuretics and cardiac glycosides.
Zusterzeel et al. [16]	United States	Meta-analysis	2014	4076 (22%)	Women with LBBB benefited from CRT-D at a shorter QRS duration than men with LBBB.

ACE = angiotensin-converting enzyme; ADRs = adverse drug reactions; ARBs = angiotensin receptor blockers; CMR = cardiac magnetic resonance; CRT-D = cardiac resynchronization therapy-devices; CVD = cardiovascular disease; EDV= end-diastolic volume; HPDs = hypertensive disorders; HF = heart failure; HFpEF = heart failure with preserved ejection fraction; LBBB = left bundle branch block; LV = left ventricle; LVEF = left ventricle ejection fraction; MRA = aldosteron receptor antagonist; RCT = randomized controlled trial; RV = right ventricle; SGLT2i = sodium-glucose cotransporter-2 inhibitors.

**Table 2:** Knowledge gaps in clinical practice guidelines and recommendations.

Topic	Gaps in Evidence
Heart failure classification	
LVEF thresholds	Sex-based threshold values in LVEF
NPs levels	Relevance of sex and hormonal status in establishing The normal reference range for NPs
Sex-specific risk factors	
HDPs and APOs	Prognostic role of preeclampsia/eclampsia
Premature menopause	HRT and SERMs
Cardiomyopathies	Treatment of PPCM and inflammatory cardiomyopathies
Heart failure management	
Guideline-directed medical therapy	Sex-specific therapeutic options and drug dosages
CIED	Sex-specific QRS duration thresholds

APOs = adverse pregnancy outcomes; CIED = cardiac implantable electronic device; CMP = cardiomyopathy; HF = heart failure; HPDs = hypertensive disorders; HTR = hormone replacement therapy; LVEF = left ventricle ejection fraction; NPs = natriuretic peptides; PPCM = peripartum cardiomyopathy; SERMs = selective estrogen-receptor modulators.

## Data Availability

Not applicable.
